# Extended trochanteric osteotomy: comparison of 3 modes of fixation: metallic wires, cables, plate, about a series of 157 cases

**DOI:** 10.1051/sicotj/2018017

**Published:** 2018-06-13

**Authors:** Jean Louis Prudhon, Nicolas Tardy

**Affiliations:** Centre osteo articulaire, 5 rue des tropiques, 38130 Echirolles France

**Keywords:** Extended trochanteric osteotomy, Fixation, Metallic wires, Cables, Plate

## Abstract

*Introduction*: The trans femoral osteotomy was initially described by Wagner in 1987 and the extended trochanteric osteotomy (ETO) was described by Younger et al. in 1995 and is considered to be the gold standard technique for removal of well-fixed femoral stems in revision total hip arthroplasty (THA). The purpose of this report is:

- to compare the different types of fixation metallic wires, cables, metallic reinforcement plate (MRP) we have used in revision THA where an ETO was performed;
- analyse the clinical and radiological outcomes of these devices at 1 year;
- analyse the complication.

*Material* *and method*: It is a retrospective continuous monocentric series of 157 patients where an ETO was performed. It was fixed by an MRP in 17 patients, cables in 43, metallic wires in 97. The main outcome was the consolidation of the osteotomized femoral flap (OFF). Secondary outcomes were Postel Merle d’Aubigne score and complications occurred at 1 year follow up. Qualitative variable was presented as percentage, quantitative variables as mean or median, standard deviation and range.

*Result*: 157 patients (73−46, 5% females) were included. Mean age at surgery was 66.7 year (sd = 10.63). Mean interval between index surgery and revision was 11.07 year (sd = 5.67). Causes for revision and bone defects were comparable. At 1 year OFF is healed without displacement in 82% with metallic wires, 70% with cables, 88% with MRP. Not significant.

*Discussion*: Fixation of the femoral flap is a technical issue in ETO. Metallic wires and cables are the most commonly used to secure the fixation. Fixation with a metallic plate is reported in a few number of articles and may be helpful specially when a fracture of the OFF occurred during surgery.

## Introduction

As the number of revision total hip arthroplasty (THA) is regularly growing, several technical issues had risen and have been solved by orthopaedic surgeons. Extended trochanteric osteotomy (ETO) is one of these solutions proposed to remove femoral implant in order to improve surgical technique as well as the clinical outcomes.

The trans femoral osteotomy was initially described by Wagner [1] in 1987 and the ETO was described by Younger et al. [2] in 1995 and is considered to be the gold standard technique for removal of well-fixed femoral stems. Its indications for use can be applied to nearly any patient undergoing a femoral revision. It is the investigator’s preference to employ this technique when revising patients with thin, osteoporotic proximal femoral cortical bone or if there is any curved remodelling deformity of the proximal femur.

There are several pitfalls that the surgeon should aim to avoid when performing an ETO:

- meticulous haemostasis;
- the preservation of the soft tissue attachments of the vastus lateralis and gluteus medius (minimize Trendelenburg gait), maintain the blood supply of the osteotomy fragment;
- care should be taken to protect the osteotomy fragment from intraoperative fracture, particularly when applying cerclage cables [3];
- even in experienced hands, ETO can still result in complications such as non-union (1.3–1.6%), fracture (2.4–4%), superior migration of osteotomy fragment (1.2–6.7%), and reoperation (2.7–10.2%) [4,5];
- patient status has also to be considered in this challenging surgery.

In most of the cases, the revision is required 10–15 years after index surgery. Severe comorbidities, age, intraoperative complications, length of surgery are significant factors that may alter clinical results [6].

ETO provides good exposure of the previous implant and adequate access to the canal for cement removal, cleaning of the endo-medullary bone, reaming and preparation of new implant insertion even in septic revision [7]. However, the overall complication rate has been reported as high as 24% [5]. Non-union or proximal migration of the osteotomy fragment can occur [8–10], whereas the dorsal portion particularly of the proximal femur is, vulnerable to intra-operative split fractures.

**Figure 1:**
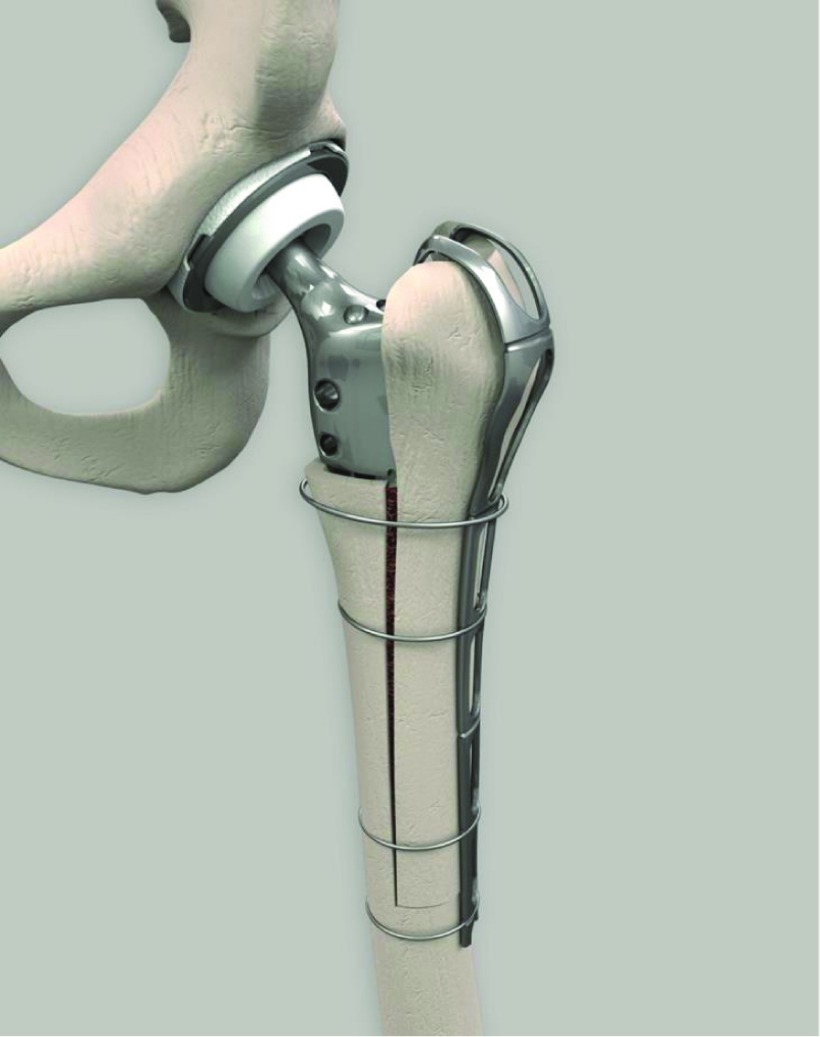
Metallic reinforcement plate (MRP) Integra^R^.

Even though the surgical technique has been safely performed with respect of these criteria’s, in some cases where the bone conditions are critical, it is very difficult to get a reliable and solid fixation of the femoral flap. The risk of non-union is high. In order to avoid a scratch of the remaining lateral cortex and to improve the fixation of the femoral slot a specific openwork metallic reinforcement plate (MRP) so called “INTEGRA PLATE” has been designed, with 2 proximal hooks, that can be fixed with metallic wires (Figure 1).

The purpose of this report is

- to compare the different types of fixation (metallic wires, cables, MRP we have used in revision THA where an ETO was performed.
- analyse the clinical and radiological outcomes of this device at 1 year.
- analyse the complication.

## Material and methods

### Surgical technique

Pre-operative planning is one of the major steps of the procedure. X-ray standard evaluation must include a frontal standing pelvic view, a frontal and sagittal view of the entire femur, a full lower limb standing X-ray. CT scan is mandatory to evaluate osteolytic lesions, status of the cortical bone, soft tissue, muscle and granuloma.

We have to determine:

- what significant surgical issues we have to deal with during the surgery? (fixation of the previous implant, level of the cement mantle, distal migration of cement in the medullary canal, length of the previous implant, osteolytic lesions or distal femoral cortex thickening);
- what length is the most suitable to easily remove the femoral implant, secure the distal fixation of the new implant.

**Figure 2:**
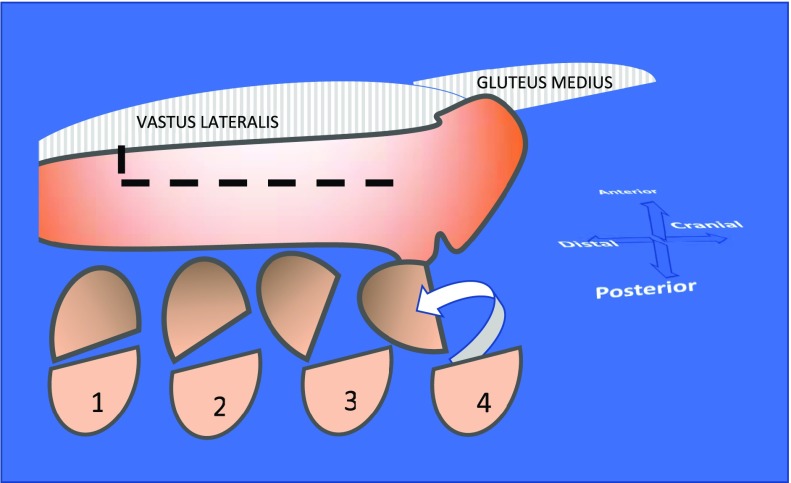
Original Wagner’s approach.

**Figure 3:**
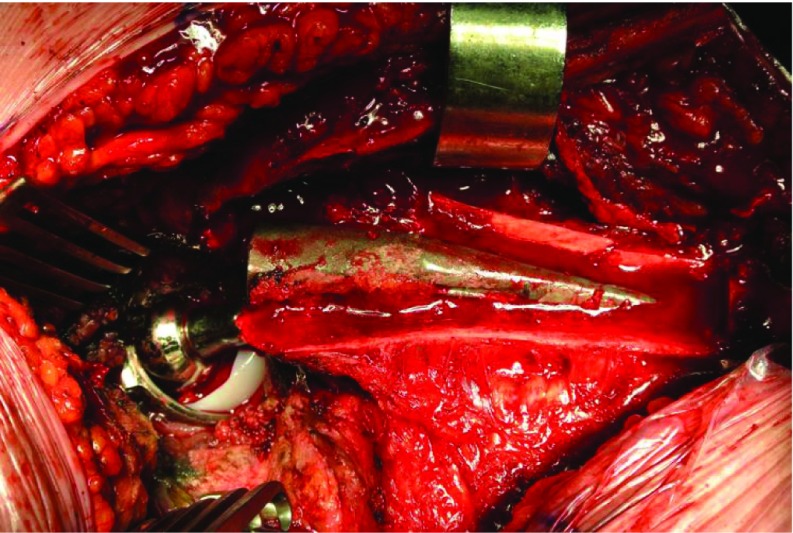
ETO performed to remove a well fixed femoral component implanted in excessive retroversion.

**Figure 4:**
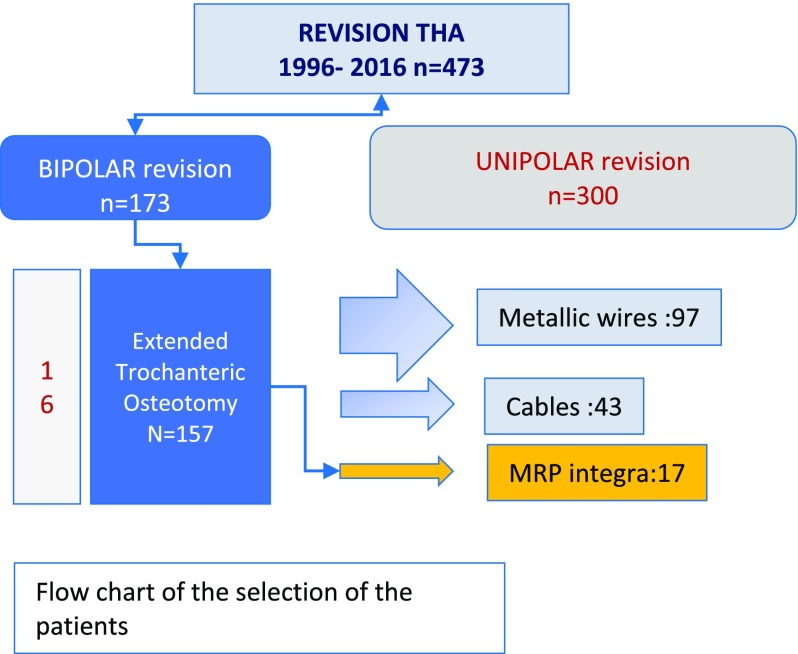
Flow chart of the selection of the patients.

Patient is lying on a strict lateral decubitus position. A long skin incision is performed according to the length of ETO; the hip is approached from proximal to distal. The hip joint is opened posteriorly. The femoral shaft is exposed with an anterior splitting of the vastus lateralis. Its trochanteric insertion remains intact. The level of the distal bone cut is measured and identified thanks to two 3.5 mm drilled holes. The osteotomy includes half of the femoral shaft. It starts posteriorly from distal to proximal femur. The anterior osteotomy is performed from distal to proximal as far as we can go without injuring the muscles. An osteotome is blindly introduced anteriorly to cut the anterior cortex. The last cut is the distal cut. The osteotomised femoral fragment (OFF) is retracted anteriorly with the vastus lateralis (Figures 2 and 3). If the previous implant is cemented it is generally easy to separate the femoral cortex and the implant. If the previous implant is a well-fixed cement less component, this manoeuver can be critical. Removal and cleaning of cement debris, granulomas, membranes is a long and meticulous step as well as preparation of the host bone for the new implant. The ETO facilitates all these steps and decreases the risk of perforation or mal positioning of the implants. The fixation of the OFF is prepared before implanting definitive devices. A variable number of wires are passed around the medial cortex. When acetabular component is implanted, femoral component and bearing are relocated, the metallic wires are tightened. When the lateral cortex is good quality looking, the fixation with 3 or 4 wires is generally sufficient. But when the cortical bone is thin, partially fractured or destroyed, reduction and fixation is a real technical issue. That is why we have designed the MRP Integra plate to be applied on the lateral cortex with 2 hooks getting in the great trochanter (GT). The length of the plate can be adapted. It is fixed with metallic wires. The windows of the plate preserve blood supply and allow bone grafting (Figure 4).

### Design features (Figure 1)

The MRP is made of stainless steel. It is 250 mm long, 1.5 mm thick with 2 hooks to grab GT and abductor muscles. Its shape can be conformed to the bone due to the reduced thickness of the device. This small thickness is a mandatory contraindication to its use as a plate to fix a shaft fracture. It is only an additional device to improve reduction and fixation of an ETO. MRP is manufactured by groupe Lépine TM Genay (France).

In this series metallic wires were AO type (Protek Zimmer), cables were Dall Milles cables (Zimmer) (Figures 6 and 7).

### Characteristics of the series

**Figure 5:**
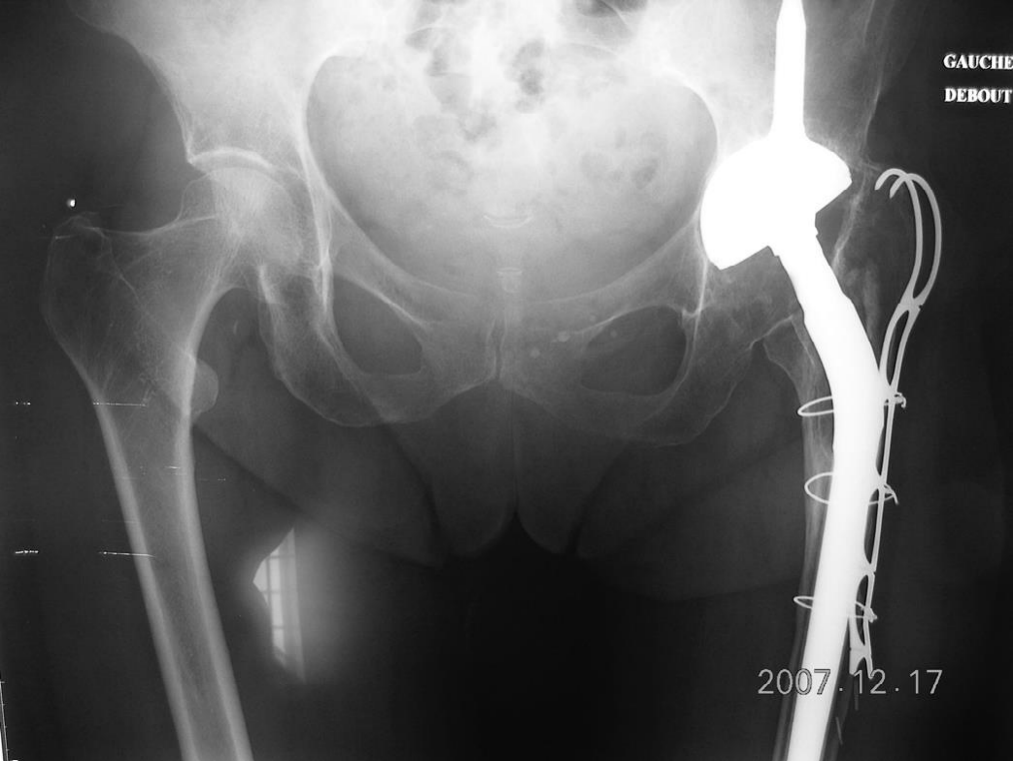
ETO fixed by MRP Integra, complete healing of the osteotomised fragment at 1 year.

**Figure 6:**
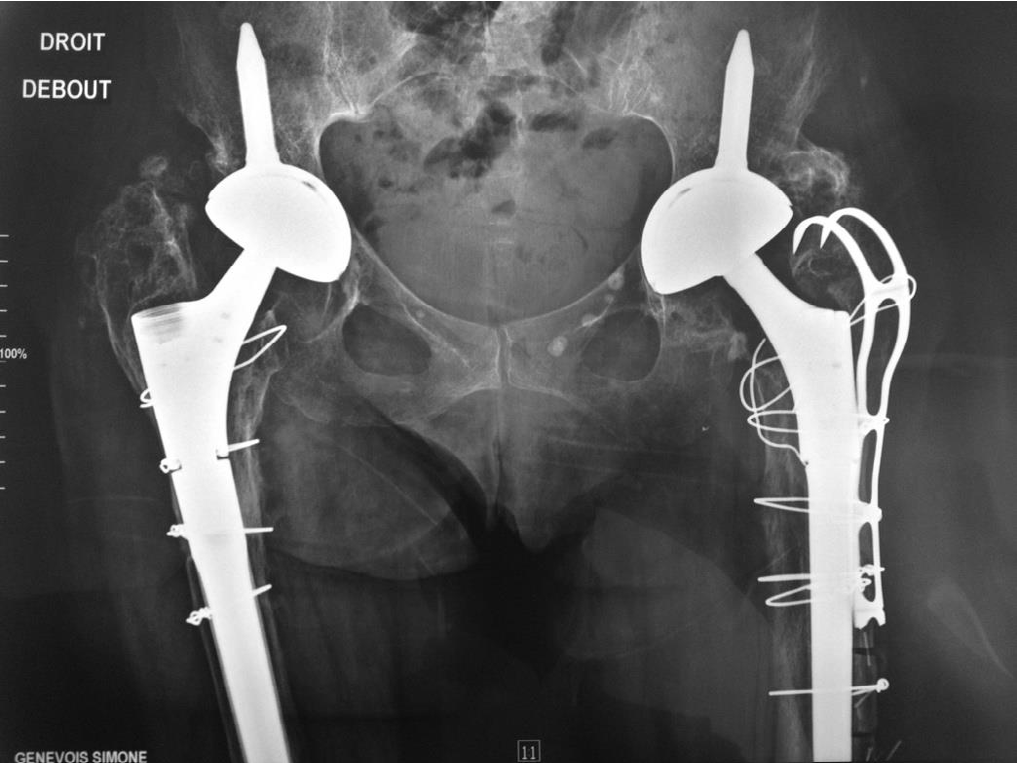
Bilateral bipolar revision. ETO fixed by metallic wires (right) and MRP Integra (left), complete healing of the osteotomised fragment at 1 year on both sides.

**Figure 7:**
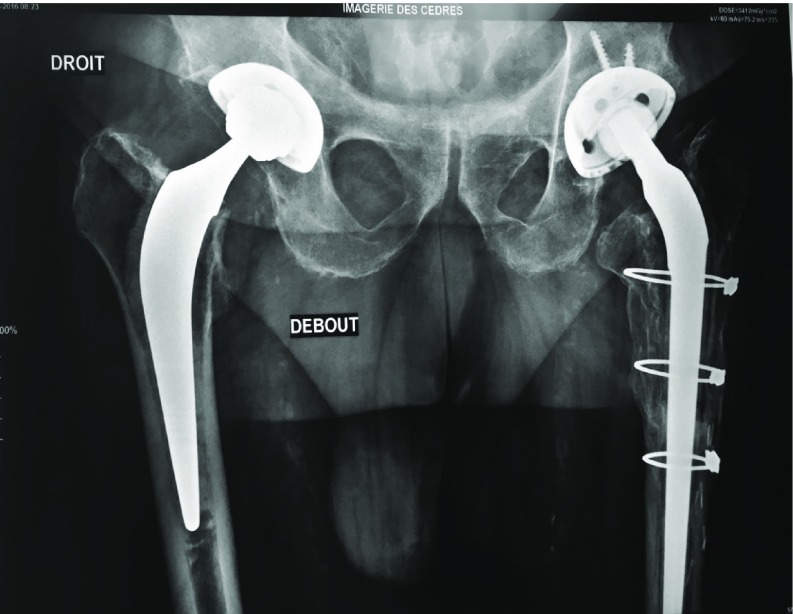
ETO fixed by cables.

Among 473 revisions THA we have extracted 157 patients were an ETO was performed. In this monocentric cohort, the ETO was fixed by MRP in 17 patients, cables in 43, metallic wires in 97 (Figures 5–7).

### Data collection

Data were retrospectively collected from our data base File Maker Pro. Patient characteristics, reasons for revision, surgical details, Postel Merle d’Aubigne (PMA) score [11], Femoral defects (AAOS Classification [12], SoFCOT classification, complications, clinical and X-ray evaluation at 1 year were collected by the author.

All the patients were evaluated at 1 year to assess clinical and radiological outcomes.

Migration or subsidence of the femoral implant, complications were noted as well as consolidation of the femoral flap with 3 degrees:

- bone union without displacement (Figure 5);
- bone union with cranial migration of the flap;
- non-union.

### Statistical analysis

The comparison of qualitative variables was performed by Chi-square test (or Fisher’s exact test according to validity conditions). The comparison of quantitative variables was performed by Sample *t*-test. Significance threshold was set up at 0.05. Qualitative variable was presented as percentage, quantitative variables as mean or median, standard deviation and range.

## Results

### Demography (Table 1)

157 cases of ETO were performed from 1998 to 2009. Seventeen patients were operated on with ETO and MRP, 97 patients were operated on with metallic wires and 43 were operated on with cables.

Overall there were 76 females (48.4%) with a mean age of 66.5 years old. The groups were comparable in terms of mean age, sex ratio, interval between surgery and reason for revision. Regarding bone quality score, the distribution is a bit more heterogeneous with more severe cases (AAOS grade C and D) in the group MRP, the less severe bone condition being the metallic wire group.

**Table 1:** Demographic data.

	MRP (Mean ± SD)	Metallic wires	Cables
*N*	17	97	43
Female/male	9 (53%)/8 (47%)	49(51%)/48(49%)	18(42%)/25(58%)
Age at revision	66.3 ± 10.83 Range 50–79	67.2 ± 10.5 Range 40–86	66 ± 9.74 Range 44–82
Interval between initial surgery and revision	12.9 ± 5.6 Range 4–20	11.6 ± 5.4 Range	9.8 ± 5.4 Range 1–22
Previous cemented component	13 (76.4%)	78 (80.4%)	38 (88.4%)

Reason for revision			
Aseptic loosening	14 (82.4%)	69 (71.1%)	36 (83.7%)
Sceptic loosening	0	4 (4.4%)	3 (7.9%)
Implant breakage	1 (5.8%)	4 (4.4%)	0
Peri-prosthetic fracture	1 (5.8%)	8 (8.8%)	2 (4.6%)
Hip fusion	1 (5.8%)	3 (3.3%)	0
AAOS	A1a = 1	A1a = 17	A1a = 7
	A1b = 1	A1b = 5	A1b = 0
	A2 = 1	A2 = 3	A2 = 1
	B = 1	B = 10	B = 5
	C = 10	C = 27	C = 29
	D = 3	D = 12	D = 1
SOFCOT	Grade 1 = 0	Grade 1 = 11	Grade 1 = 6
	Grade 2 = 5	Grade 2 = 25	Grade 2 = 2
	Grade 3 = 9	Grade 3 = 25	Grade 3 = 17
	Grade 4 = 3	Grade 4 = 29	Grade 4 = 18

### Follow-up

At 1-year post op, all the 17 patients with MRP were reviewed by the author. In the other groups, 3 patients of the metallic wire group and 1 patient of the cable were deceased. Two patients of the metallic wire group were lost to follow-up. Therefore 151 patients were reviewed at 1 year.

### Primary outcome (Table 2)

Stability of the OFF fixation was recorded during surgery and after immediate post op X-rays. Fixation was considered as solid in 14 cases, precarious in 2, in 1 case the femoral slot broke during surgery.

**Table 2:** Comparison of the different fixation modes in ETO.

	MRP	Metallic wires	Cables
*N*	17	97	43
Healed in position	15 (88%)	80 (82%)	30 (70%)
Healed with displacement	0	7 (7%)	7 (16%)
Not healed	2 (12%)	6 (6%)	5 (12%)
Revision before 1 year	0	3 (2%)	0
Deceased	0	3 (2%)	1 (1%)
Lost to follow up	0	2 (1%)	0

There are no significant differences between the 3 types of fixation regarding the consolidation of the OFF (*p* = 0.14).

In the MRP group, the OFF was considered as healed without displacement in 15 cases and not healed with displacement in 2 cases, of which one had a breakage of MRP and wires. Hardware removal was necessary but no additional surgery was proposed to fix the OFF.

In the metallic wires group (97 cases) 6 (6%) OFF did not heal at 1 year.

In the cables fixation group (43 cases) 5 (12%) OFF did not heal at 1 year.

### Secondary outcome (Table 3)

Preoperative PMA ROM score was slightly better for the metallic wire group.

All the 3 groups had improvement of the PMA score whatever the category (pain, ROM and ability to walk). The mean gain of PMA score for MRP was 2.5 for pain, 2.5 for mobility and 2 for walking ability. The mean gain of PMA score for metallic wires group was 2.68 for pain, 1.73 for mobility and 2.79 for walking ability. The mean gain of PMA score for cables group was 2.67 for pain, 2.37 for mobility and 2.4 for walking ability. Differences were not significant.

**Table 3:** Clinical scores.

PMA scrore	MRP	Metallic wires	Cables
Preoperative PMA (Mean ± SD)			
Pain	2.4 ± 0.79	2.49 ± 0.86	2.35 ± 0.95
ROM	2.5 ± 0.72	3.34 ± 0.85	2.74 ± 1.1
Ability to walk	2.3 ± 0.68	2.36 ± 0.89	2.48 ± 1.01
Postoperative PMA (Mean ± SD)			
Pain	4.9 ± 0.82	5.17 ± 0.86	5.02 ± 0.99
ROM	5 ± 0.56	5.07 ± 0.92	5.11 ± 1.05
Ability to walk	4.3 ± 1.04	5.15 ± 0.93	4.88 ± 1.21

### Complications (Table 4)

No infection occurred in the group MRP, 1 patient had a dislocation of the hip prosthesis treated by a closed reduction under general anaesthesia. No recurrence occurred.

**Table 4:** Complications.

	MRP	Metallic wires	cables
*N*	17	97	43
Dislocation	1 (5.8%)	7 (7.2%)	1 (2.3%)
Delayed wound infection	2 (11%)	3 (3.1%)	0
Hematoma	–	3 (3.1%)	2 (4.7%)
Infection	–	3 (3.1%)	2 (4.7%)
Deep veinous thrombosis	–	4 (4.1%)	3 (7.0%)
Revision within the 1st year	–	4 (4.1%)	0

Two patients had a delayed wound healing that finally solved at 2 months without further surgery.

One patient had a breakage of the MRP. The OFF initial fixation was considered as precarious with an intra operative fracture of the lateral cortex. The OFF did not heal with a cranial displacement. The plate and the broken wires were removed after 24 months without any attempt to fix the OFF. The patient was limping but was able to walk 2 blocks with a crutch.

No major revision (revision of at least one of the components) has been reported.

The main complications in the 2 other groups were infection (2 (4.6%) in cable group and 3 (3.1%) in wire group), dislocation (7 (7.2%) in wire group and 1 (2.3%) in cable group). Four patients (4.1%) of the cable group were revised within the first year.

## Discussion

### Complexity of THA revision

As far as the number of revision THA is growing accordingly to the increase number of primary THA, technical issues are getting more frequent and difficult. These complex situations have to take into account, the patient, the indication for revision, the local bone and soft tissue conditions. In our series mean age of the patients at revision surgery was 66.3 year.

The mean interval between index surgery and revision was 11.07 year (sd = 5.67).

Most of the THA were revised for aseptic loosening with poor bone quality (13 AAOS grade C and D).

### Extended trochanteric osteotomy (when, how, why)

Since the first description by Wagner in 1987 the Wagner osteotomy became in 1995 the so-called ETO. More than 80 publications referring to this technique can be found in PubMed. We have been using this technique since 1991 on 157 cases of revision THAs.

Most of the time the decision is made pre-operatively. This technique is mandatory needed in the cases where a severe curvature of the upper femur is observed, in implant breakages, in distal migration of the cement mantle, in femoral cortex thickening at the tip of the implant. The length of the osteotomy is depending upon the length of the previous implant and the difficulties to remove a well-fixed implant either cemented or cement less.

The ETO minimises the risk of femoral perforation and fracture, in particular in the deformed femur [13]. Preservation of an intact muscular-osseous-sleeve comprised of the gluteus medius and minimus, the GT, and the vastus lateralis, allows physiological reconstruction of the hip’s soft tissue envelope and prevents proximal migration of the osteotomy.

The preservation of the muscles and the soft tissue is a favourable factor for blood supply [14]. The ETO may save time compare to the endo-medullary techniques. It also prevents from intra-operative fractures, allows a complete removal of granuloma, large debridement of the necrotic bone especially useful when treating septic loosening [7].

### OFF fixations in ETO

To fix the OFF, we have used metallic wires in 97 cases, cables in 43 cases, MRP in 17 cases. At 1 year follow up the status of the OFF according to the type of fixation is presented in Table 2. There are no significant differences between the 3 types of fixation (Figure 7, Table 2).

Several options of fixation are reported in the literature. Most of them compare fixation with metallic wires or cables or cords [15–17].

There are only 2 publications of fixation with a plate as we propose. Zhu et al. [15] report a cadaveric study of different types of fixation (wires, wire with a short claw plate, long claw plate). Sheridan et al. [18] reported on the use of ETO in the treatment of 31 femoral stem revision in peri-prosthetic fractures. Nineteen of these were fixed using cables only, and 11 were fixed using a cable-plate construct. The cable-plate construct performed better than cables alone.

Our series is a retrospective nonrandomized monocentric study. Sample size is small, but the use of a reinforcement plate has to be considered when femoral lateral cortex is thin and sore and the risk of breakage high when tightening the wires.

## Conclusion

THA revision is a challenging surgery for the patient and the surgeon. Over 3 decades many improvements have been observed in implant manufacturing, surgical techniques, patient selection in order to improve the outcomes. However, it remains a complex and difficult surgery with many local or general complications. ETO has been a significant advance in this field of Hip revision surgery. Fixation of the OFF is a real issue. MRP is a reliable and helpful technique and its results are consistent with that of reported with the other techniques.

## Conflict of interest

Jean Louis Prudhon is consultant for groupe Lépine and receives royalties from groupe Lépine.
